# Optic Neuropathy following Botulinum Toxin Injection into the Medial Rectus Muscle for Diplopia

**DOI:** 10.18502/jovr.v17i2.10806

**Published:** 2022-04-29

**Authors:** Mohammad Reza Khalili, Shahla Hosseini, Mohammad Shirvani, Maryam Sadat Sadati

**Affiliations:** ^1^Poostchi Ophthalmology Research Center, Shiraz University of Medical Sciences, Shiraz, Iran; ^2^Department of Dermatology, Shiraz University of Medical Sciences, Shiraz, Iran

**Keywords:** Botulinum Toxin, Diplopia, Optic Neuropathy

## Abstract

**Purpose:**

To report a case of optic neuropathy (ON) following botulinum toxin A (BTA) injection into the medial rectus muscle.

**Case Report:**

We describe a 37-year-old man with unilateral ON after a BTA injection into the left medial rectus for treatment of traumatic sixth nerve palsy. Oral prednisolone was prescribed for 14 days. After two weeks, his visual acuity returned to 20/20.

**Conclusion:**

Botulinum toxin-induced neuropathy is a rare and vision-threatening complication of BTA. In patients with recent injection of BTA who present with visual complaints, botulinum toxin-induced neuropathy should be considered.

##  INTRODUCTION

Botulinum toxin is a polypeptide that immunologically includes seven subtypes. Subtype A is the most common type used in ophthalmology. This neurotoxin inhibits the release of vesicles containing acetylcholine in the neuromuscular synaptic cleft leading to chemo-denervation.^[1,2]^ It can also block the pain receptors and inhibit secretion of nor-epinephrine in the soft tissue.^[2,3]^ Its effects typically initiate three to four days after injection and lasts for three to four months.^[1,2][3]^ In addition to widespread cosmetic purposes, it has several therapeutic applications including for relief of migraine headache, hemi-facial and blepharospasm, strabismus, lid retraction and restrictive myopathy associated with thyroid eye disease (TED), and neuropathic pain.^[1,2][3][4][5][6]^ Common strabismus conditions that have been managed by using botulinum toxin A (BTA) injection are infantile esotropia (ET), intermittent exotropia, sixth nerve palsy, fourth nerve palsy, thyroid-related orbitopathy (TRO), and nystagmus.^[3,5]^ Optic neuropathy (ON) associated with BTA injection is very rare. Herein, we describe a case of ON following a BTA injection into the medial rectus (MR) for management of horizontal binocular diplopia.

##  CASE REPORT

**Figure 1:**
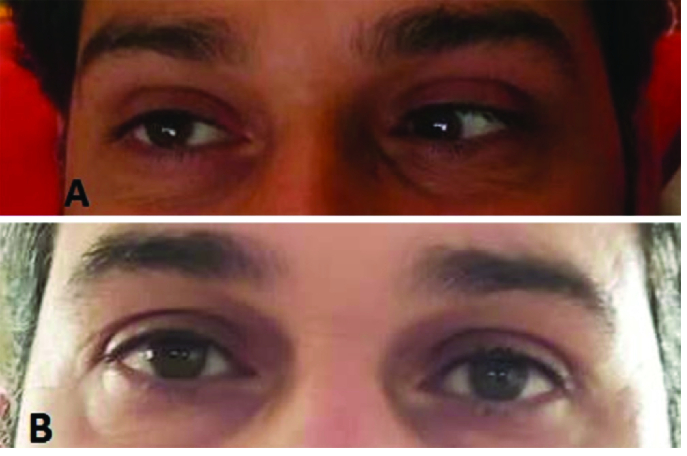
External eye photos in primary gaze position, at presentation (A) showed left eye esotropia, and three month later (B) showed no deviation.

**Figure 2:**
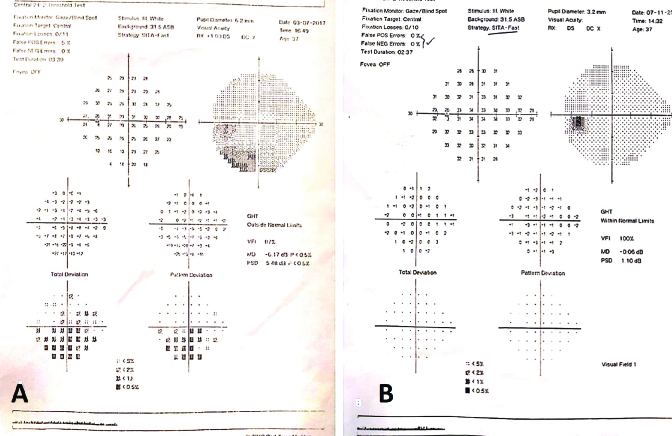
Automated visual field test (24-2 threshold) of the left eye; at presentation (A) reveals inferior visual field defect, and three months later (B) shows complete recovery without scotoma.

**Figure 3:**
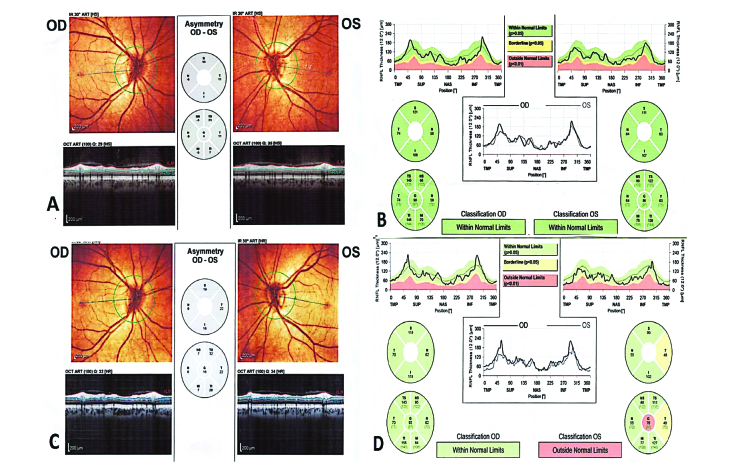
Peripapillary optical coherence tomography; at presentation, the optic nerve head images (A), and retinal nerve fiber layer thickness maps (B) were normal in both eyes. Three months after presentation, the optic nerve head image showed mild temporal pallor in the left eye (C), and retinal nerve fiber layer thickness maps demonstrated a reduction in the temporal retinal nerve fiber layer thickness of the left eye (D).

**Figure 4:**
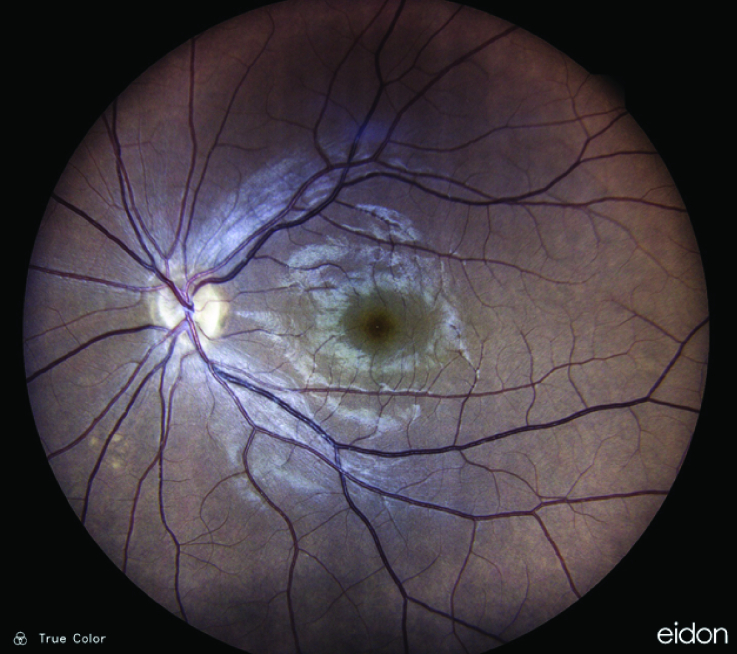
Color fundus photo of the left optic nerve three months after presentation shows mild temporal disc pallor.

A 37-year-old man who was a taxi driver presented with a complaint of blurred vision, especially in the inferior visual field (VF) and dyschromatopsia in his left eye (LE) which started one day prior to presentation. He had a motor-cycle accident 25 days before his presentation. After trauma, the ophthalmic examinations had revealed best-corrected visual acuity (BCVA) of 20/20 in both eyes (monocular and binocular visual acuity), negative relative afferent pupillary defect (RAPD), normal fundus and optic nerve appearance, normal color vision and confrontation VF tests, and a limitation of abduction in the LE. Imaging studies of his brain and orbit were unremarkable. Two days prior to his referral to us while in another center he had undergone an injection of BTA into the left MR (DysportⓇ with a dose of 15 units) because of intolerable horizontal binocular diplopia due to traumatic sixth nerve palsy. Before the BTA injection, the ophthalmic examinations had shown BCVA of 20/20 in both eyes (monocular visual acuity testing), negative RAPD, normal fundus and optic nerve appearance, normal color vision and VF tests, and a 25 prism diopters ET in the LE. At the initial visit, his BCVA was 20/30 in the LE and 20/20 in the right eye (RE). The intraocular pressure measurements were 11 mm/Hg in the LE and 13 in the RE. Pupillary examination showed positive RAPD in the LE. External eye examination revealed limitation of abduction in the LE without any signs of proptosis, ptosis, and ecchymosis. Ocular motility examination revealed limitation of abduction, and alternate cover test demonstrated 18 prism diopters ET in the LE [Figure 1A]. Color vision test (Ishihara color plates) was abnormal (with 4 correct responses out of 12 plates) in the LE and was normal in the RE. The Humphrey VF test was normal in the RE and demonstrated inferior hemi-field scotoma (altitudinal defect) in the LE [Figure 2A]. Fundus examination revealed healthy retina and optic disc in both eyes. The optic nerve head optical coherence tomography (peripapillary retinal nerve fiber layer [RNFL] analysis) [Figures 3A and 3B] and the fluorescein angiography of the optic nerve were within normal range in both eyes. Visual evoked potential (VEP) testing showed decreased P100 amplitude and normal latency in the LE and was normal in the RE. Magnetic resonance imaging of the orbit and brain was normal. The Color-Doppler sonography of the carotid arteries and echocardiography was normal. Complete blood count, erythrocyte sedimentation rate, C-reactive protein, prothrombin time, partial thromboplastin time, antinuclear antibody, and angiotensin-converting enzyme were within normal ranges. The patient was diagnosed with ON associated with BTA injection. Oral prednisolone at a dosage of 75 mg/day (1 mg/kg/day) was initiated and continued for 14 days and tapered during the second week. Two weeks following medical therapy, RAPD was negative and BCVA increased to 20/20. At the three-month follow-up examination, the BCVA was 20/20 in both eyes, the VF defect disappeared [Figure 2B], the color vision test was normal, and the RAPD was still negative. In primary position, the patient had no deviation [Figure 1B] but the alternate cover test showed a 4 prism diopter ET in the LE. The fundus examination revealed mild temporal disc pallor [Figure 4], and RNFL optical coherence tomography demonstrated a reduction in the RNFL thickness in the temporal disc area [Figures 3C and 3D].

**Table 1:** Data of previous case reports of botulinum toxin-induced neuropathy


**Author (yr)**	**Age(gender) /underlying disease**	**Laterality (OD/OS)**	**Indication /site of injection**	**Injected dose of botulinum toxin**	**Time of presentation**	**First visual acuity and visual field pattern**	**Treatment and final outcome**
**Y Chun^[7]^ (2017)**	43(F)/–	OD	Cosmetic /masseter muscles, to the area with the greatest cross-sectional surface of both masseter bellies at three points	25 units (75 units per side) (BOTOX)	4 hr after injection	BCVA of 20/800 inferior visual field defect	Pulse of IV methylprednisolone (3 day) and oral prednisolone (7 day) Final BCVA of 20/20 with small para-central scotoma after two months
**YH Chen^[8]^ (2010)**	54(F)/Sjogren's syndrome	OS	Cosmetic/glabella and frontal (BOTOX and hydrogel injection)	Not mentioned	Immediately after injection	BCVA of 20/70 and superior hemi-field scotoma	Combination therapy with oral steroid, IV antibiotics and aspirin Third nerve palsy resolved 17 days later, BCVA increased to 20/30 and mild paleness of inferior optic disc
**BS.Korn ${}^{4}$ (2006)**	64(F)/Graves' disease	OD	Diplopia associated with TRO/ right medial rectus and right inferior rectus muscles	15 units (0.3 mL) in the right medial rectus and 15 units (0.3 mL) in the right inferior rectus muscles (BOTOX)	3 weeks after injection	BCVA of 20/60 and inferior altitudinal field defect	Oral prednisone and orbital decompression surgery Final BCVA of 20/30
	53(F)/ Hypothyroidism from 10 years ago	OS	Vertical diplopia associated with TRO/left inferior rectus muscle	10 units (0.2 mL) (BOTOX)	3 weeks after injection	Abnormal color vision and para-central visual field defect	Oral prednisone and orbital decompression surgery Reduction in 2 mm proptosis
	61(F)/Graves' disease	OD	Significant diplopia associated with TRO/right and left medial rectus and right inferior rectus muscles	10 units (0.2 mL) in the right medial rectus, 10 units (0.2 mL)in the left medial rectus and 5 units (0.1 mL) in the right inferior rectus muscles (BOTOX)	3 weeks after injection	BCVA of 20/25 and inferior para-central visual field depression	Oral prednisone and then radiotherapy of right orbit and three-wall orbital decompression surgery Reduction in 9 mm proptosis
	
	

##  DISCUSSION 

Several ophthalmic complications were reported following cosmetic and therapeutic injections of BTA, including ptosis, strabismus, diplopia, dry eye, tearing, mydriasis, acute close angle glaucoma, globe perforation, and ischemic retinopathy.^[1,5,7,8,9]^ The present report described a patient who developed ON following a BTA injection into the MR muscle for management of diplopia. The most important differential diagnosis for ON in this patient was traumatic optic neuropathy (TON). Although TON also manifests with acute vision loss, a positive RAPD and abnormal color vision and/or alteration in the VF test, the vision loss usually manifests immediately following trauma and is often severe (20/400 or less). In TON, the optic disc becomes atrophic within 8–12 weeks and the generalized reduction of the peripapillary RNFL thickness usually occurs within 2–12 weeks after trauma.^[10,11]^ We excluded TON as a probable cause of vision loss because the visual complaint of our patient started immediately after the BTA injection and ophthalmic examinations including visual acuity, optic disc appearance, RAPD test, color vision and VF tests after trauma and before the BTA injection procedure were all completely normal. In addition, the optic disc did not show diffuse atrophy and generalized reduction of the peripapillary RNFL thickness in the examination 12 weeks after the trauma. Following the BTX injection, in addition to the existence of diplopia, the patient started to complain of blurred vision and VF defect, and monocular visual acuity and the VF testing confirmed it. Oral prednisolone was started for the patient and at the three-month follow-up examination, the BCVA improved to 20/20. However, the optic nerve head optical coherence tomography (OCT) demonstrated a mild reduction in the RNFL thickness in the temporal disc area (RNFL thickness in the temporal region was 63 µm at presentation and 48 µm at the three-month follow-up). There are five reported cases of unilateral botulinum toxin-induced ON in the literature [Table 1].^[4,7,8]^ In our study, ON occurred two days after administering the injection into the patient's MR muscle. In previous reports, ON occurred three weeks after the injection of BTA into the extraocular muscles in three patients with TED,^[4]^ 4 hr after the injection into the masseter muscle,^[7]^ and immediately after the injection of BTA with Polyacrylamide Hydrogel used for esthetic purposes.^[8]^ These time differences may suggest that various mechanisms can underlie botulinum toxin-induced ON.

Young Chun et al have suggested that BTA induced-thrombosis occurs by stimulation of the vascular endothelial cells and by platelet aggregation. In addition, retrograde migration of localized thrombus which is formed secondary to vascular wall damage may lead to ischemic neuropathy and retinal artery occlusion in the early hours following the injection.^[7]^ Yi-Hsing Chen et al have mentioned reflux of BTA to the periorbital arteries which causes peripapillary arteries vasospasm, resulting in optic nerve hypo-perfusion immediately after the injection.^[8]^ Korn et al have proposed that compression of the optic nerve might occur through several mechanisms including intramuscular hematoma, increased volume of the orbit, and disfiguration of the extraocular muscle. As they mentioned, toxic effects of BTA on the optic nerve secondary to leakage of BTA from the site of the injection can also induce ON several weeks after the injection. Of note, compressive ON resulted from enlargement of orbital muscles and fat and subsequent orbital congestion in the natural course of TED was another explanation for presentation of ON in their report.^[4]^

The recommended dose of BTA for injection into the MR muscle for management of sixth nerve palsy is 2.5–7.5 units (BOTOXⓇ).^[12,13]^ In our patient, 15 units of BTA (DysportⓇ) that is approximately equal to 6 units of BOTOX had been injected into the MR. Korn et al have used 10–15 units of BTA (BOTOXⓇ) for management of TRO [Table 1].^[4]^

Although Kutluk et al reported that intraocular injection of botulinum toxin had no significant effect on the retinal cells of rabbits, Berliocchi et al suggested that botulinum toxin C led to synaptic disarray and stimulation of apoptosis and inflammation *via* its proteolytic effects on the neuronal cells according to the VEP and electro-retinography findings.^[14,15]^ In our patient because of the acute presentation of ON, the occurrence of toxic ON, and/or ischemic ON is the probable diagnosis. Therefore, during the BTA injection into the extraocular muscles, in order to reduce the diffusion of the toxin into the surrounding tissues, an accurate dosing of BTA, the proper needle size, an appropriate injection site, and a very gentle injection should be considered.^[6]^

In previous reports, on presentation, two cases had optic nerve head swelling and three had normal optic disc without any swelling.^[4,7,8]^ In our case, the optic disc examination was normal at the initial visit, but three months later, mild temporal pallor of the optic nerve was evident. These findings reveal that ON associated with BTA may occur in the anterior or posterior (retro-bulbar) part of the optic nerve. Hence, a normal optic disc examination at the initial presentation cannot rule out the possibility of ON, and additional assessments including VEP, VF testing, color vision evaluation, and consultation with a neuro-ophthalmologist are recommended.^[4,7,8]^

Several patterns of VF abnormalities were demonstrated in previous reports of botulinum toxin-induced ON. Two cases had inferior visual hemi-field defect, similar to our case.^[4,7]^ Other cases had superior scotoma,^[8]^ para-central scotoma, and inferior para-central depression.^[4]^

Because different mechanisms underlie botulinum toxin-induced ON, management and treatment of this condition is varied. In previous cases, oral prednisone and/or intravenous methylprednisolone have been the initial treatment. We treated our patient with oral prednisolone for two weeks with a good response. It was proposed that corticosteroids might reduce botulinum toxin-induced inflammation and decrease the direct damage by the toxin on the neuronal cells of the optic nerve. Management of the underlying conditions and complications such as TRO and cellulitis is also necessary.^[4,5,7,8]^ In our patient, ON occurred without any underlying condition.

The present report emphasized the consideration of ON in every patient who presented with visual function problems (e.g., blurred vision, VF defects, and dyschromatopsia) following cosmetic or therapeutic BTX injections. Systemic corticosteroids as described might contribute to the management of BTX- induced ON.

In summary**, **this report described a very rare case of ON following the administration of a BTA injection into the MR for management of diplopia that occurred in a healthy young patient. The patient was managed with oral prednisolone. Complications of the BTA injection should be explained to patients before administering the injection. Ophthalmologists should also be aware of these complications, especially when being used in therapeutic injections into the extra-ocular muscles.

##  Declaration of Patient Consent

The authors certify that they have obtained all appropriate patient consent forms. In the form the patient has given his consent for his images and other clinical information to be reported in the journal. The patient understand that his name and initial will not be published and due efforts will be made to conceal his identity, but anonymity cannot be guaranteed.

##  Financial Support and Sponsorship

Nil.

##  Conflicts of interest

The authors declare that they have no conflict of interest.
